# The Effectiveness of Clinical Pharmacist-Led Consultation in the Treatment of Infectious Diseases: A Prospective, Multicenter, Cohort Study

**DOI:** 10.3389/fphar.2020.575022

**Published:** 2020-09-08

**Authors:** Jiaxing Zhang, Xiaosi Li, Rui He, Wenyi Zheng, Joey Sum-wing Kwong, Ling Lu, Tianyi Lv, Rong Huang, Mei He, Xiaoyan Li, Xue Wang, Qin Fang, Lingyu Wei, Yang Liu, Shuya Chen, Xiaogai Qin, Juan Xie

**Affiliations:** ^1^ Department of Pharmacy, Guizhou Provincial People’s Hospital, Guiyang, China; ^2^ Department of Pharmacy, Hospital of Chengdu Office of People’s Government of Tibetan Autonomous Region, Chengdu, China; ^3^ Experimental Cancer Medicine, Department of Laboratory Medicine, Karolinska Institute, Stockholm, Sweden; ^4^ Clinical Research Center and Center of Allogeneic Stem Cell Transplantation(CAST), Karolinska University Hospital Huddinge, Stockholm, Sweden; ^5^ Global Health Nursing, Graduate School of Nursing Science, St. Luke’s International University, Tokyo, Japan; ^6^ Department of Pharmacy, The Second People’s Hospital of Guiyang, Guiyang, China; ^7^ Department of Pharmacy, Xingyi People’s Hospital, Xingyi, China; ^8^ Department of Pharmacy, Qian Xi Nan People’s Hospital, Xingyi, China; ^9^ Department of Pharmacy, The First People’s Hospital of Bijie City, Bijie, China; ^10^ Department of Pharmacy, Tongren Municipal People’s Hospital, Tongren, China; ^11^ Department of Pharmacy, The People’s Hospital of Qiannan, Duyun, China; ^12^ Department of Pharmacy, The Affiliated Hospital of Guizhou Medical University, Guiyang, China; ^13^ Department of Pharmacy, Guizhou Cancer Hospital, Guiyang, China; ^14^ Department of Pharmacy, The Second Affiliated Hospital of GuiZhou Medical University, Kaili, China; ^15^ Department of Pharmacy, The First People’s Hospital of Guiyang, Guiyang, China; ^16^ Department of Pharmacy, Affiliated Wudang Hospital of Guizhou Medical University, Guiyang, China

**Keywords:** Antimicrobial Stewardship Program, clinical pharmacist-led consultation, infectious diseases, pharmaceutical service, cohort study

## Abstract

**Background:**

Antimicrobial resistance (AMR) is a serious global health threat and leads to a huge challenge to infectious diseases (ID) treatment. To tackle AMR, regional ‘Antimicrobial Stewardship Programs’ (ASP) have been implemented in many countries. Due to insufficient clinical pharmacy resources, a major intervention mode of ASP in China is through clinical pharmacist-led consultation (CPC). The current study aims to prospectively evaluate this intervention and compare the effectiveness of CPC served by ID and non-ID clinical pharmacists.

**Methods:**

We conducted a prospective and multicenter cohort study based on a regional registry database in 17 hospitals in Western China, including consecutive patients with ID between April 2017 and December 2019. Baseline characteristics including sex, age, liver and kidney function, comorbidity, infection severity were prospectively collected and recorded. The main exposure of interest was whether the attending physician adopted recommendations of the clinical pharmacist in the therapeutic scheme. The outcome was the infection effective response, assessed during day 3–7 after completing CPC. Multivariate analyses were performed by generalized linear mixed models.

**Results:**

A total of 2,663 ID patients were included in the final analysis according to the predesigned inclusion and exclusion criteria. The number of patients whose treatment followed and did not follow the pharmacists’ suggestion was 2,529 and 134, respectively. CPC intervention could improve the ID patient prognosis in the context of other confounders controlled (*Adjusted Odds ratio*(*AOR*)=1.838, 95%*Confidence Interval*(*CI*)=[1.212, 2.786]), and the effectiveness of CPC served by ID and non-ID clinical pharmacists might be equivalent (*AOR*=0.958, 95%*CI*[0.740, 1.240]). Special consultation (*AOR*=1.832, 95%*CI*[1.106, 3.035]) and surgical treatment of infectious sites (*AOR*=1.380, 95%*CI*[1.039, 1.834]) had positive influences on the patient prognosis, while hypoalbuminemia (*AOR*=0.694, 95%*CI*[0.523, 0.921]), liver dysfunction (*AOR*=0.705, 95%*CI*[0.559, 0.889]), presence of high-risk factors (*AOR*=0.775, 95%*CI*[0.613, 0.980]), and increased infection severity (*AOR*=0.631, 95%*CI*[0.529, 0.753])were associated with a decrease in effective response rate, independently.

**Conclusion:**

This study suggests that CPC is a promising pharmacist-led intervention to improve ID treatment, and it can achieve standardization among clinical pharmacists with different backgrounds by some measures. Policy/decision-makers should promote this intervention mode in developing countries or regions where there is an insufficient number of clinical pharmacists.

## Introduction

Antimicrobial resistance (AMR) has become a serious global health challenge that can undo the decades of progress in declining morbidity and mortality from infectious diseases (ID) (Alsan et al., 2015; Marston et al., 2016; Thakur and Gray, 2019). AMR caused approximately 700,000 death per year worldwide (O’Neill, 2016), and the mortality from resistant infection was predicted to increase to 10 million by 2050 (Thakur and Gray, 2019). With the rise of AMR, the cumulative cost against this issue was nearly 100 trillion USD (O’Neill, 2016). Developing countries are currently the major consumers of antibiotics and thus have the grimmest situation of AMR (Laxminarayan and Chaudhury, 2016; Gebretekle et al., 2018). Several reports have indicated high presence of methicillin-resistant S. *aureus* in different healthcare settings of developing countries (e.g., Morocco (14.4%), Ivory Coast (16.8%), Kenya (27.7%), Nigeria (29.6%), Ethiopia (42.8%), South Africa (52%), and Cameroon (72%)) (Hayat et al., 2020). A report based on the data from the China Antimicrobial Resistance Surveillance System (CARSS) and the China Antimicrobial Surveillance Network (CHINET) indicated that gram-negative bacilli have higher antimicrobial resistance profiles than gram-positive bacilli. Besides, the prevalence of Carbapenem-resistant Klebsiella pneumoniae (CRKP) in China showed a remarkable increase from 2005 to 2017 (Hu et al., 2018). Due to the knowledge gaps of both public (Li et al., 2020) and medical professionals such as pharmacists in antibiotics (Hayat et al., 2019), the irrational use of antimicrobial has amplified the AMR burden of China which is one of the world’s largest producers and consumers of antibiotics (Hayat et al., 2019; Shi et al., 2020).In 2014, the World Health Organization (WHO) warned of a “*post-antibiotic era, in which common infections and minor injuries can kill* (World Health Organization, 2014).” Ever since then, the “Antimicrobial Stewardship Program” (ASP) has been successively implemented in many countries (Charani et al., 2019). ASP is instituted to promote the appropriate use of antimicrobials, improve patient outcome, reduce microbial resistance, and decrease the spread of infection caused by multidrug-resistant organisms (Charani et al., 2019). Meanwhile, the inclusion of clinical pharmacists in ASP has been shown to result in significant improvement in terms of the quality of antibiotic use from various aspects, such as improving guideline-concordant antimicrobial prescribing (Smith et al., 2018; Wang et al., 2018; Fay et al., 2019; Bishop et al., 2020), lessening the emergence of multidrug resistance (Li et al., 2017), shortening the days of antibiotic therapy (Wirtz et al., 2020), reducing the hospital stay of patients (Sadyrbaeva-Dolgova et al., 2020), and decreasing patients mortality (Li et al., 2017).

ASP in China was initiated in 2011 with the promulgation of the Guideline for Clinical Application of Antimicrobial Agents (GCAAA), which required clinical pharmacists to participate in the management of antimicrobials, particularly in special antibiotics (vancomycin, carbapenem, tigecycline). Unfortunately, due to the limited number of clinical pharmacists, not every department of Chinese hospitals have enough clinical pharmacists, particularly ID clinical pharmacists. So, clinical pharmacist-led consultation (CPC), which depended on the collaboration of both ID and non-ID pharmacists, was gradually developed and became an integral and essential part of ASP (Zhang et al., 2019a; Zhang et al., 2019b; Zhang et al., 2020a).

With the implementation of ASP, more attention has been paid to the role of CPC in ID treatment. A previous systematic review (Zhang et al., 2019a) including 50 case series found that, in China, CPC with an excellent acceptance rate (93.60%) was an effective intervention for ID treatment. However, the methodological quality of the included studies was compromised due to high risk of selection and reporting bias. Well-designed prospective cohort studies are thus urgently needed. In another single-center cohort study (Zhang et al., 2019b), researchers demonstrated that CPC could improve the prognosis of ID patients. However, the conclusions drawn in the study should be further verified due to the limited representativeness of patients. Therefore, this multicenter cohort study based on a registry database was conducted to evaluate the effectiveness of CPC in ID treatment on patient outcomes and potential determinants.

## Materials and Methods

We followed the STROBE (STrengthening the Reporting of OBservational studies in Epidemiology) Statement for the conduct and reporting of this cohort study (**Supplementary Table S1**).

### Study Design and Setting

This prospective, multicenter, cohort study was carried out in 17 hospitals across Guizhou Province, China (the Guizhou Provincial People’s Hospital (Principal Investigator and Lead Institution), the First People’s Hospital of Guiyang, the Second People’s Hospital of Guiyang, the First People’s Hospital of Bijie City, Tongren Municipal People’s Hospital, Xingyi People’s Hospital, Qian Xi Nan People’s Hospital, People’s Hospital of Anshun City, the People’s Hospital of Qiannan, Guizhou Cancer Hospital, the Affiliated Hospital of Guizhou Medical University, the Second Affiliated Hospital of Guizhou Medical University, the Affiliated Baiyun Hospital of Guizhou Medical University, Affiliated Wudang Hospital of Guizhou Medical University, Guiyang Maternal and Child Health Care Hospital, the People’s Hospital of Sansui, and the People’s Hospital of Xifeng). The study was approved by the Ethics Committee of the Guizhou Provincial People’s Hospital (2017066, **Supplementary Tables S2** and **S3**) and conducted in accordance with the Declaration of Helsinki.

### Study Population

Inpatients with confirmed diagnosis of ID (caused by bacteria, viruses, parasites or fungi) seeking CPC services from April 2017 to December 2019 were recruited consecutively upon informed consent. The following conditions formed our study exclusion criteria: (1) clinician-initiated CPC after the use of specific antibiotic regimens including the fourth-generation cephalosporins, vancomycin, teicoplanin, linezolid, carbapenems, tigecycline, echinocandins, voriconazole, and amphotericin B, in which case the clinicians requesting CPC met the requirements of a successful ASP but clinical pharmacist did not participate in the treatment of the ID patients; (2) the patient died or was discharged from the hospital before the consultation.

### Description of Consultation Intervention

The attending clinician requested CPC when treating patients with complicated ID. Upon the request, the clinical pharmacy department assigned a qualified clinical pharmacist (successful completion of 1-year residency training and certified by specialty professional board) to execute the consultation.

The assigned pharmacist comprehensively evaluated the infection of the patient in accordance with the clinical symptoms, laboratory tests, etiological and imaging examination, and provided the treatment suggestion (e.g., antibiotic type, dose, frequency, and course) based on the patient’s conditions (e.g., age, sex, liver and kidney function, underlying disease, etc.), the best clinical evidence (as informed by evidence-based practice guidelines), and the medication characteristics (antibiogram, pharmacokinetics-pharmacodynamics, and adverse events). Finally, the clinician decided whether to adopt the recommendations in the therapeutic regimen.

### Exposure Factor and Covariates

The main exposure factor was whether the clinicians adopted the clinical pharmacists’ recommendations in the therapeutic regimen, and was identified by comparing the consistency between clinicians’ prescriptions and pharmacists’ suggestions. We categorized patients whose treatment did or did not follow the pharmacists’ suggestions into the intervention group or the control group respectively. Acceptance refers to that clinicians completely or partially adopted the advices from pharmacists, and the acceptance rate (AR) was calculated using the following formula (Zhang et al., 2020b):

AR (%)=number of patients whose treatment followed pharmacists' suggestionsnumber of patients who asked for CPC×100%

In the intervention group, the background of clinical pharmacists (ID specialist versus non-specialist) was set as another exposure factor. Other covariates (age, sex, department, time of consultation after hospitalization, consultation type and purpose, number of infectious sites, febrile symptom, hemogram, serum albumin level, kidney and liver function, infection type and severity, inflammatory indicators, etiological examination results, imaging examination results, comorbidity, and surgical treatment of infectious sites) were also recorded prospectively.

### Assessment of Outcome

Effective response was defined as partial or complete resolution of clinically significant infectious signs or symptoms, improvement or resolution of computed tomography (CT) or magnetic resonance imaging (MRI) findings, and negative culture results (Zhang et al., 2019b). The follow-up period was from day 3 to day 7 after the completion of CPC.

### Data Collection and Management

A registry database (Database Management System for Clinical Pharmacist-Led Consultation for Infectious Diseases in Guizhou Province, http://39.108.6.93/webui/index.html#login) specialized for this study was set up and utilized to record and manage the patient data. The data were recorded and crosschecked by two independent investigators. To avoid detection bias, the clinical pharmacist participating in CPC could not assess the outcome.

### Statistical Analysis

Software SPSS 19.0 and Stata 14.0 were used to perform statistical analyses. The significance level was set at 0.05. Categorical variables were presented as numbers with percentages. Chi-square tests (or Fisher’s Exact tests) or Mann-Whitney U tests were used for group comparisons. A generalized linear mixed model was performed to compare the patient outcome of the intervention group with that of the control group and calculate the odds ratio (OR). This was achieved by treating the effective response as the dependent variable while the main exposure factor and other important covariates, including sex, age, department, hypoalbuminemia, kidney and liver function, consultation type, etiological evidence, imaging evidence, comorbidity, high-risk factors, surgical treatment, and infection severity, as independent variables. Considering the aggregation of data existed at the study center (hospital) level, a random coefficient model was utilized. Because all the covariates were categorical, we created a separate category (NA=Not Applicable) for missing values to include in our analyses. Then, sensitivity analyses were performed by a random intercept model or a random coefficient model involving all the covariates. The same statistical analysis methods were also used to explore whether the background of the clinical pharmacist could influence the prognosis of ID patients receiving CPC intervention.

## Results

### Studied Population

**Figure 1:**
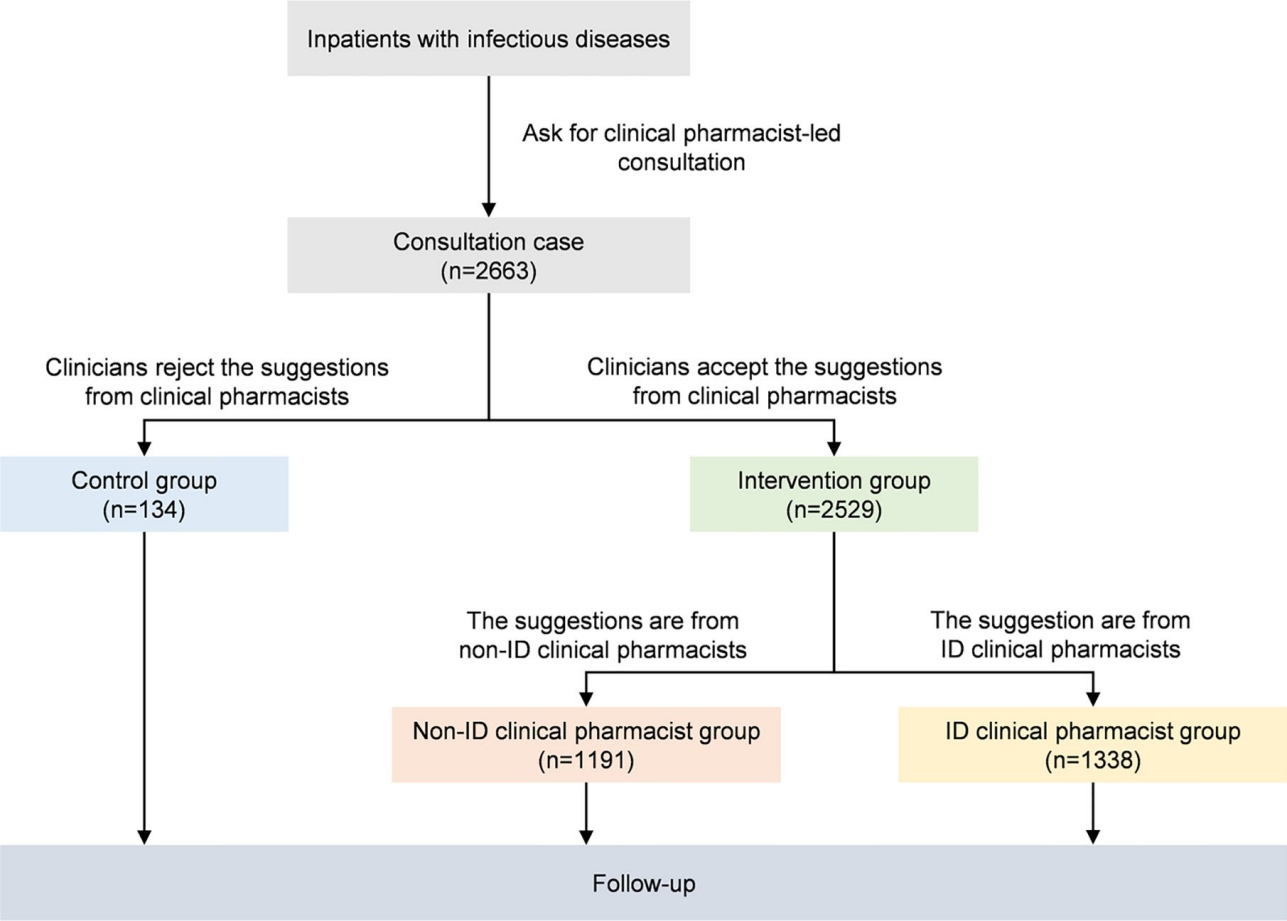
Flowchart of study patients. ID, Infectious Diseases.

A total of 2,663 patients were included in the final analysis (**Figure 1**); their baseline characteristics are presented in **Table 1**. The study patients were mainly from 18 to 65 years old (58.54%) and 57.60% were male. The number of infectious sites and infection type could not be determined in 130 (4.88%) and 294 (11.04%) patients, respectively, due to unexplained infections. As the clinicians did not apply for related examinations, information about hemogram, hypoalbuminemia, kidney function, liver function, and inflammatory indicator (C reactive protein, procalcitonin, or interleukin-6) were unavailable in 53 (1.99%), 439 (16.49%), 127 (4.77%), 117 (4.39%), 533 (20.02%) patients, respectively.

**Table 1:** Characteristics of study patients (N=2663).

	**Total** **(n=2663)**	**Control Group** **(n=134)**	**Intervention Group** **(n=2529)**	***P value***	**Intervention Group**		
					Non-ID pharmacist (N=1191)	ID pharmacist (N=1338)	*P value*
**Sex**				0.614			0.051
Female	1129 (42.40%)	54 (40.30%)	1075 (42.51%)		482 (40.47%)	593 (44.32%)	
Male	1534 (57.60%)	80 (59.70%)	1454 (57.49%)		709 (59.53%)	745 (55.68%)	
**Age group, years**				0.218			0.607
0-17	275 (10.33%)	14 (10.45%)	261 (10.32%)		118 (9.91%)	143 (10.69%)	
18-65	1559 (58.54%)	86 (64.18%)	1473 (58.24%)		684 (57.43%)	789 (58.97%)	
≥66	829 (31.13%)	34 (25.37%)	795 (31.44%)		389 (32.66%)	406 (30.34%)	
**Department**				0.570			<0.001^*^
Intensive Care Unit	251 (9.43%)	12 (8.96%)	239 (9.45%)		78 (6.55%)	161 (12.03%)	
Surgical Department	1207 (45.32%)	60 (44.78%)	1147 (45.35%)		519 (43.58%)	628 (46.94%)	
Pediatric Department	165 (6.20%)	11 (8.21%)	154 (6.09%)		72 (6.05%)	82 (6.13%)	
Oncology Department	234 (8.79%)	9 (6.72%)	225 (8.90%)		142 (11.92%)	83 (6.20%)	
Infection & Respiratory Department	154 (5.78%)	8 (5.97%)	146 (5.77%)		52 (4.37%)	94 (7.03%)	
Emergency Department	88 (3.30%)	7 (5.22%)	81 (3.20%)		46 (3.86%)	35 (2.62%)	
Traditional Chinese Medicine Department	7 (0.26%)	0 (0.00%)	7 (0.28%)		6 (0.50%)	1 (0.07%)	
Invasive Technology Department	53 (1.99%)	0 (0.00%)	53 (2.10%)		8 (0.67%)	45 (3.36%)	
Internal Medicine Department	504 (18.93%)	27 (20.15%)	477 (18.86%)		268 (22.50%)	209 (15.62%)	
**Time of consultation after hospitalized, days**				0.856			<0.001^*^
1-7	1192 (44.76%)	61 (45.52%)	1131 (44.72%)		486 (40.81%)	645 (48.21%)	
>7	1471 (55.24%)	73 (54.48%)	1398 (55.28%)		705 (59.19%)	693 (51.79%)	
**Type of consultation**				0.020^*^			<0.001^*^
General Consultation	2536 (95.23%)	133 (99.25%)	2403 (95.02%)		1161 (97.48%)	1242 (92.83%)	
Special Consultation	127 (4.77%)	1 (0.75%)	126 (4.98%)		30 (2.52%)	96 (7.17%)	
**Purpose of consultation**				0.398			0.862
Therapeutic Regimen Adjustment	2343 (87.98%)	121 (90.30%)	2222 (87.86%)		1045 (87.74%)	1177 (87.97%)	
Initial Therapeutic Regimen	320 (12.02%)	13 (9.70%)	307 (12.14%)		146 (12.26%)	161 (12.03%)	
**Number of infectious sites**				0.919			<0.001^*^
1	2106 (79.08%)	105 (78.36%)	2001 (79.12%)		910 (76.41%)	1091 (81.54%)	
>1	427 (16.04%)	23 (17.16%)	404 (15.97%)		230 (19.31%)	174 (13.00%)	
NA	130 (4.88%)	6 (4.48%)	124 (4.90%)		51 (4.28%)	73 (5.46%)	
**Febrile symptom**				0.710			0.005^*^
No	1270 (47.69%)	66 (49.25%)	1204 (47.61%)		602 (50.55%)	602 (44.99%)	
Yes	1393 (52.31%)	68 (50.75%)	1325 (52.39%)		589 (49.45%)	736 (55.01%)	
**Hemogram**				0.297			0.128
Normal	509 (19.11%)	23 (17.16%)	486 (19.22%)		209 (17.55%)	277 (20.70%)	
Abnormal	2101 (78.90%)	106 (79.10%)	1995 (78.88%)		958 (80.44%)	1037 (77.50%)	
NA	53 (1.99%)	5 (3.73%)	48 (1.90%)		24 (2.02%)	24 (1.79%)	
**Hypoalbuminemia**				0.098			0.936
No	707 (26.55%)	25 (18.66%)	682 (26.97%)		325 (27.29%)	357 (26.68%)	
Yes	1517 (56.97%)	86 (64.18%)	1431 (56.58%)		672 (56.42%)	759 (56.73%)	
NA	439 (16.49%)	23 (17.16%)	416 (16.45%)		194 (16.29%)	222 (16.59%)	
**Kidney function**				0.086			<0.001^*^
Normal	1852 (69.55%)	87 (64.93%)	1765 (69.79%)		848 (71.20%)	917 (68.54%)	
Abnormal	684 (25.68%)	44 (32.84%)	640 (25.31%)		312 (26.20%)	328 (24.51%)	
NA	127 (4.77%)	3 (2.24%)	124 (4.90%)		31 (2.60%)	93 (6.95%)	
**Liver function**				0.477			<0.001^*^
Normal	1884 (70.75%)	99 (73.88%)	1785 (70.58%)		902 (75.73%)	883 (65.99%)	
Abnormal	662 (24.86%)	32 (23.88%)	630 (24.91%)		268 (22.50%)	362 (27.06%)	
NA	117 (4.39%)	3 (2.24%)	114 (4.51%)		21 (1.76%)	93 (6.95%)	
**Inflammatory indicator**				0.288			<0.001^*^
Normal	473 (17.76%)	20 (14.93%)	453 (17.91%)		206 (17.30%)	247 (18.46%)	
Increased	1657 (62.22%)	92 (68.66%)	1565 (61.88%)		797 (66.92%)	768 (57.40%)	
NA	533 (20.02%)	22 (16.42%)	511 (20.21%)		188 (15.79%)	323 (24.14%)	
**With etiological evidence**				0.045^*^			0.231
No	1553 (58.32%)	67 (50.00%)	1486 (58.76%)		685 (57.51%)	801 (59.87%)	
Yes	1110 (41.68%)	67 (50.00%)	1043 (41.24%)		506 (42.49%)	537 (40.13%)	
**With imaging evidence**				0.004^*^			0.647
No	1278 (47.99%)	48 (35.82%)	1230 (48.64%)		585 (49.12%)	645 (48.21%)	
Yes	1385 (52.01%)	86 (64.18%)	1299 (51.36%)		606 (50.88%)	693 (51.79%)	
**Comorbidity**				0.692			0.009^*^
No	1586 (59.56%)	82 (61.19%)	1504 (59.47%)		676 (56.76%)	828 (61.88%)	
Yes	1077 (40.44%)	52 (38.81%)	1025 (40.53%)		515 (43.24%)	510 (38.12%)	
**With high risk factors of infection**				0.632			0.018^*^
No	1246 (46.79%)	60 (44.78%)	1186 (46.90%)		529 (44.42%)	657 (49.10%)	
Yes	1417 (53.21%)	74 (55.22%)	1343 (53.10%)		662 (55.58%)	681 (50.90%)	
**Surgical treatment of infectious sites**				0.460			0.011^*^
No	2057 (77.24%)	107 (79.85%)	1950 (77.11%)		945 (79.35%)	1005 (75.11%)	
Yes	606 (22.76%)	27 (20.15%)	579 (22.89%)		246 (20.65%)	333 (24.89%)	
**Type of infection**				0.896			<0.001^*^
Community-acquired infection	1667 (62.60%)	86 (64.18%)	1581 (62.51%)		722 (60.62%)	859 (64.20%)	
Hospital-acquired infection	702 (26.36%)	33 (24.63%)	669 (26.45%)		362 (30.39%)	307 (22.94%)	
NA	294 (11.04%)	15 (11.19%)	279 (11.03%)		107 (8.98%)	172 (12.86%)	
**Severity of infection**				0.628			0.553
Mild	452 (16.97%)	30 (22.39%)	422 (16.69%)		172 (14.44%)	250 (18.68%)	
Moderate	1388 (52.12%)	60 (44.78%)	1328 (52.51%)		665 (55.84%)	663 (49.55%)	
Severe	823 (30.91%)	44 (32.84%)	779 (30.80%)		354 (29.72%)	425 (31.76%)	
**Effective Response**				< 0.001^*^			0.896
Yes	2147 (80.62%)	90 (67.16%)	2057 (81.34%)		970 (81.44%)	1087 (81.24%)	
No	516 (19.38%)	44 (32.84%)	472 (18.66%)		221 (18.56%)	251 (18.76%)	

Data are n (%). Control group: patients in whom treatment regimens did not follow clinical pharmacist’s recommendations; Intervention group: patients in whom treatments adhere to clinical pharmacist’s recommendations; ID, infectious diseases; ^*^=indicated P ≤0.05; NA, Not Applicable.

The number of patients whose treatment regimens did or did not follow the pharmacists’ suggestions was 2,529 and 134, respectively, giving a total AR of 94.97%. **Table 1** shows that no significant differences existed in the majority of baseline characteristics between the intervention group and the control group except the consultation type, imaging evidence from X-ray, CT or MRI, and the etiological evidence from cultures of sputum, bronchoalveolar lavage fluid, blood, secretion, abscess, urine, cerebral spinal fluid, ascites, bone, synovial fluid, hydrothorax, and so on. The proportion of special consultations such as multiple disciplinary team consultations in the intervention group was higher than that in the control group (4.98% vs. 0.75%, *P*=0.020). Nevertheless, the percentage of patients with specific etiological or imaging evidence in the intervention group was lower than that in the control group (41.24% vs. 50.00%, *P*=0.045; 51.36% vs. 64.18%, *P*=0.004, respectively).

Among the intervention group (**Table 1**), the baseline characteristics between ID (n=1,338) and non-ID (n=1,191) pharmacist intervened group were different by the department, consultation type, time of consultation after hospitalized, kidney and liver function, comorbidity (e.g., cardiac disease, diabetes, hypertension, cancer, chronic obstructive pulmonary disease, coronary atherosclerosis, stroke, etc.), febrile symptom, inflammatory indicator, number of infectious sites, infection type, presence of high-risk factors of infection, and surgical treatment of infectious sites.

### Effective Response

**Figure 2:**
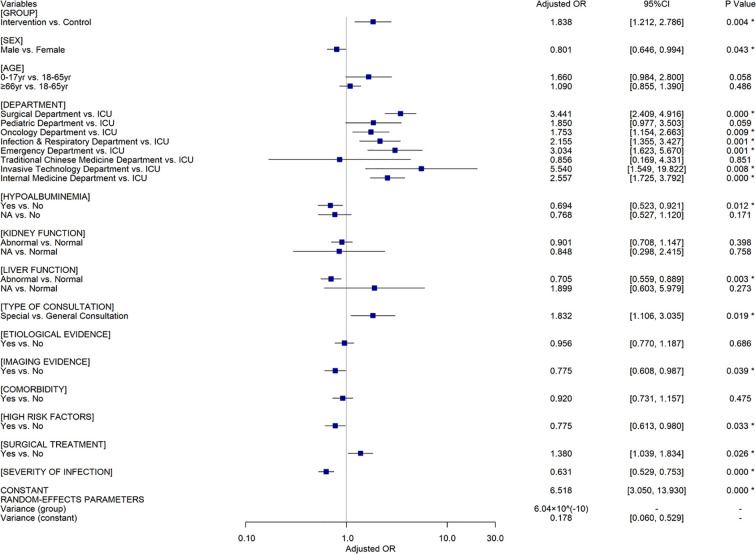
The results of multivariate analyses using a random coefficient model for all the included patients (n=2,663). Control group, patients in whom treatment regimens did not follow clinical pharmacist’s recommendations. Intervention group, patients in whom treatments adhere to clinical pharmacist’s recommendations. OR, Odds Ratio; CI, Confidence Interval; ^*^=indicated P ≤0.05; yr, years; NA, Not Applicable.

Among all included patients, 2,147 (80.62%) showed effective response. Univariate analyses demonstrated that CPC intervention, sex, department, consultation type and purpose, hypoalbuminemia, kidney and liver function, comorbidity, presence of imaging evidence, febrile symptom, hemogram, inflammatory indicator, number of infectious sites, presence of high-risk factors of infection, infection severity, and surgical treatment of infectious sites were associated with patient outcomes respectively (**Supplementary Table S4**). The effective response rate of the intervention group was significantly higher than that of the control group (81.34% vs. 67.16%, *Odds Ratio* (*OR*)=2.131, *Confidence Interval* (*CI*) = [1.466, 3.097], *P*<0.001). Considering the strong correlation between infection severity and number of infectious sites, febrile symptom, hemogram, and inflammatory indicator, infection severity instead of other covariates was input into the multivariate analyses model. Two-level random coefficient model (**Figure 2**) implies that CPC intervention, sex, department, consultation type, hypoalbuminemia, liver function, presence of imaging evidence, high-risk factors of infection, surgical treatment, infection severity are independent factors associated with the effective response of ID patients. CPC intervention (*Adjusted OR* (*AOR*)=1.838, 95%*CI*=[1.212, 2.786], *P*=0.004), special consultation (*AOR*=1.832, 95%*CI*[1.106, 3.035], *P*=0.019), surgical treatment of infectious sites (*AOR*=1.380, 95%*CI*[1.039, 1.834], *P*=0.026) had positive influences on the patient prognosis, while male patients (*AOR*=0.801, 95%*CI*[0.646, 0.994], *P*=0.043), hypoalbuminemia (*AOR*=0.694, 95%*CI*[0.523, 0.921], *P*=0.012), liver dysfunction (*AOR*=0.705, 95%*CI*[0.559, 0.889], *P*=0.003), presence of specific imaging evidence (*AOR*=0.775, 95%*CI*[0.608, 0.987], *P*=0.039), presence of high-risk factors (*AOR*=0.775, 95%*CI*[0.613, 0.980], *P*=0.033), and increased severity of infection (*AOR*=0.631, 95%*CI*[0.529, 0.753], *P* < 0.001) were associated with a decrease in effective response rate independently. Compared to the prognosis of patients in the intensive care unit (ICU), it was much better in other general wards except for the Traditional Chinese Medicine department. The results of sensitivity analyses using a random intercept model (**Supplementary Table S5**) and a random coefficient model involving all the covariates (**Supplementary Table S6**) also postulated that CPC intervention could improve the effective response of ID patients when other confounders were controlled.

**Figure 3:**
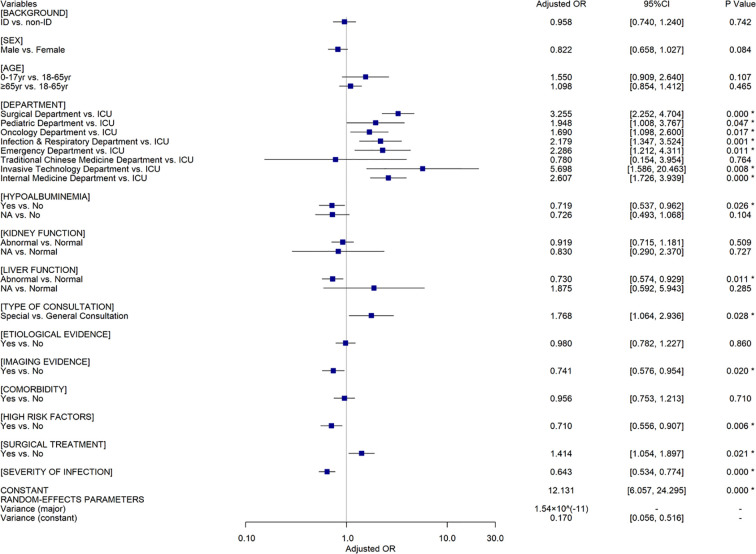
The results of multivariate analyses using a random coefficient model for the patients in the intervention group (n=2,529). OR, Odds Ratio; CI, Confidence Interval; ID, infectious diseases; ^*^=indicated P ≤0.05; yr, years; NA, Not Applicable.

Among patients in the intervention group, univariate analyses (**Supplementary Table S7**) indicated that patient outcomes were also associated with all the factors mentioned above, except for CPC intervention which is not available under this setting. 1087 and 970 ID patients attained effective response in ID and non-ID pharmacist intervened groups respectively (81.24% vs. 81.44%, *OR*=0.987, 95%*CI*[0.807, 1.206], *P*=0.896). The two-level random coefficient model (**Figure 3**) illustrates that there is no significant difference between ID pharmacist and non-ID pharmacist intervention groups (*AOR*=0.958, 95%*CI*[0.740, 1.240], *P*=0.742) in the context of other confounders controlled, which is consistent with the results of the sensitivity analyses (**Supplementary Tables S8** and **S9**).

## Discussion

This prospective, multicenter, cohort study included 2,663 patients from 17 hospitals in Guizhou Province in China to evaluate the effectiveness of CPC in ID treatment and explore the influence of clinical pharmacist´s background on the prognosis of ID patients receiving CPC intervention. We found that: (1) CPC intervention can improve the ID patient prognosis when other confounders are controlled; (2) the effectiveness of CPC delivered by ID and non-ID clinical pharmacist might be equivalent in the treatment of ID; (3) factors including sex, department, consultation type, surgical treatment, serum albumin level, liver function, specific imaging evidence, high-risk factors of infection, and infection severity are associated with the outcome of ID patients.

A previous survey (Zhang et al., 2020b) proposed that hospital rank/status could have an impact on the therapeutic efficacy of ID due to the difference in medical resources and disease severity among hospitals. In this study, we also found the aggregation of individual data at the level of hospital, which could be explained by the similarity of diagnostic capability, therapeutic technology, nursing condition, and medical resources in the same hospital. To resolve this problem, a two-level random coefficient model was utilized to perform the multivariate analysis, and sensitivity analyses were then conducted to verify the robustness of results.

The excellent AR (94.97%) in the study is consistent with a recent survey (AR=94.83%, 95%*CI*[92.95%, 96.76%]) in Guizhou Province (Zhang et al., 2020b), suggesting that CPC for ID has been well accepted by clinicians in this region. Furthermore, CPC was proved to be a promising pharmacist-led intervention in improving ID treatment in this study (*AOR*=1.838, 95%*CI*[1.212, 2.786]), which was indeed in line with findings from a previous meta-analysis (*Risk Ratio* (*RR*)=2.08, 95%*CI*[1.41, 3.06]) (Zhang et al., 2019a), a cross-sectional survey (*RR*=6.49, 95%*CI*[2.84, 14.82]) (Zhang et al., 2020b), and a single-center cohort study (*AOR*=1.738, 95%*CI*[1.028, 2.940]) (Zhang et al., 2019b). During the process of consultation, clinical pharmacists participated in the treatment of ID by discussing with clinicians and patients, communicating with microbiologists and nurses, developing a therapeutic scheme, adjusting the dosage regimen according to pharmacokinetic-pharmacodynamic results and monitoring adverse events. Therefore, it is clear that clinical pharmacists are able to assist clinicians with treatment optimization, leading to improved patient outcomes and effective use of healthcare resources.

**Figure 4:**
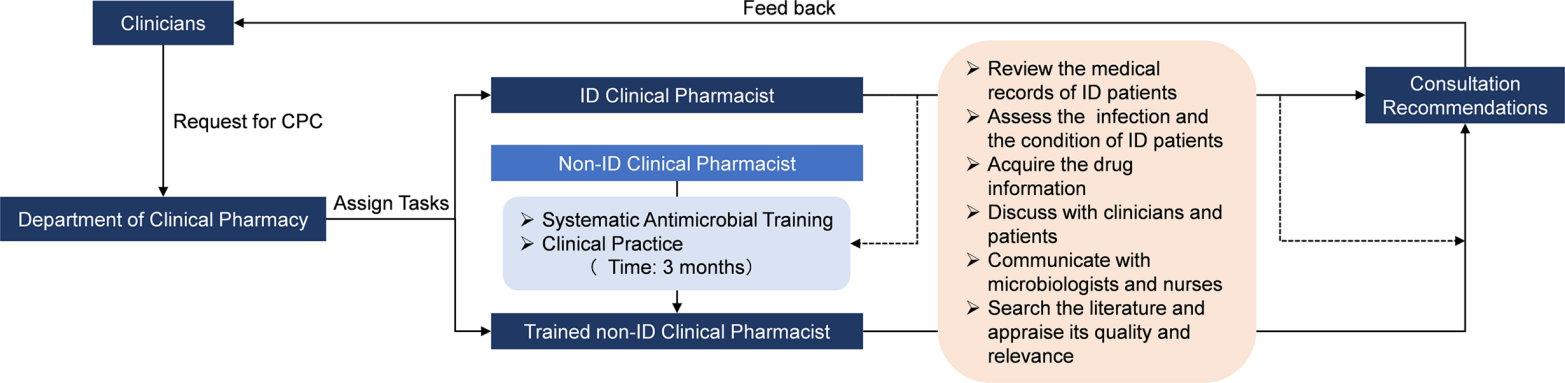
The frame of CPC mode in our institution for ID. CPC, Clinical pharmacist-led consultation; ID, Infectious diseases. The dash lines represent that the activity was guided or supervised by ID clinical pharmacist.

A recent study (Bessesen et al., 2015) concluded that ASP with dedicated ID pharmacists contributed to better adherence to the recommended antimicrobial therapy practices than that with general ward pharmacists. Nevertheless, this study showed that the background of the clinical pharmacist did not significantly alter the patient outcome in the intervention group (*AOR*=0.958, 95%*CI*[0.740, 1.240]). The CPC experience from our institution (Guizhou Provincial People’s Hospital) suggests that non-ID clinical pharmacists with appropriate educational background, systematic antimicrobial training, and rich experience in clinical practice are competent for anti-infectious consultation, and their capabilities of consultation for ID are not inferior to the ID clinical pharmacists. The Guizhou Province is a relatively less-developed region in Western China, where medical and human resources are lacking. A recent survey (Zhang et al., 2020b) indicated that the deficiency in ID clinical pharmacists was universal in most hospitals and had restricted the development of CPC for ID in Guizhou. Therefore, as the leader of clinical pharmacy in the region, our team are devoted to sharing our CPC experience with and disseminating our CPC mode (**Figure 4**) to other hospitals in Guizhou. To promote the implementation of CPC for ID, improve the quality of CPC, and narrow the consultation service gap between ID and non-ID clinical pharmacists, we have taken a series of measures during the past two years, including training clinical pharmacists, conducting continuing education courses, establishing telemedicine platforms, and designating senior clinical pharmacists to assist the grassroots medical institutions. Furthermore, we are planning to develop a clinical decision support system for CPC in ID treatment on the basis of our consultation experience, the best evidence, the prognosis prediction model of ID patients and artificial intelligence technology (e.g., knowledge graph, data mining, natural language processing, etc.) to facilitate the standardization of the consultation service among clinical pharmacists with different backgrounds in the grassroots medical institutions. A cross-sectional nationwide survey (Abubakar and Tangiisuran, 2020) among Nigerian tertiary hospitals reported that the scarcity of education and training in AMR and ID was the major barrier to pharmacists’ involvement in ASP. Therefore, we believe that it would be important to share our experience of CPC with other developing countries or regions confronting the similar challenge (Katoue et al., 2014; EI Hajj et al., 2016; Salim et al., 2016; Bilal et al., 2017).

The current study revealed that the prognosis of ID patients was associated with a number of factors. Compared to general consultations, special consultations involving clinical pharmacists and professionals from various backgrounds and disciplines (i.e., ID specialists, microbiologists, radiologists, nutritionists, and surgeons) can provide more comprehensive therapeutic suggestions and achieve a better effect (*AOR*=1.832, 95%*CI*[1.106, 3.035]). The outcome of ID patients may benefit from removal of infectious sites by surgical treatment (*AOR*=1.380, 95%*CI*[1.039, 1.834]). The infection and condition of patients in ICU are usually more complicated and severe than those in general wards, so the prognosis of patients in ICU is worse. Poor nutritional status, presence of high-risk factors (e.g., immunocompromised condition, long-term steroid exposure, with implants, hyperthermia with chills, old age, etc.) and increased severity of infection are closely aligned with the poor outcome of ID patients (*AOR*=0.694, 95%*CI*[0.523, 0.921]; *AOR*=0.775, 95%*CI*[0.613, 0.980]; *AOR*=0.631, 95%*CI*[0.529, 0.753], respectively). Besides its direct influence on the prognosis of ID patients (*AOR*=0.705, 95%*CI*[0.559, 0.889]), liver dysfunction would limit the use of many intensive antibiotics, such as tigecycline, and make the anti-infection treatment more difficult.

Randomization method was not applied in this observational study, therefore the imbalance of baseline characteristics between the intervention and control group might cause the selection bias. A randomized controlled trial is undoubtedly the gold-standard design to evaluate intervention effectiveness. However, it is impossible to randomly allocate patients to consultation or non-consultation group in Chinese hospitals due to ethical concerns. We designed and executed this prospective, multicenter, cohort study based on a country-wide, population-based registry. Eligible patients were divided into two groups: intervention group and control group, according to the main exposure factor. The hypothesis is that only if the clinician adopts the recommendation from the clinical pharmacist, the patient can really receive the CPC intervention. To minimize the influence of selection bias on the study results, we enrolled patients consecutively, improved the representativeness of patients by the multicenter design, and controlled the confounders with the multilevel model. Although we tried to control some important factors by the multivariate analysis, those unknown or undetermined factors associated with the treating outcomes of ID yet existed. In addition, this study was based on a registry which did not represent the whole of China, and it may reduce the generalizability of conclusions.

The crucial role of pharmacists in rational use of antimicrobial agents has been acknowledged in the healthcare systems of numerous countries (Northey et al., 2015; Waters, 2015; Zhou et al., 2015; Brink et al., 2016; Ellis et al., 2016; Okada et al., 2016). However, the value of CPC in ID treatment is still underestimated in China, and the lack of support from hospital administrators is the most common barrier to implementing CPC. Nevertheless, with the development of clinical pharmacy and the growth of clinical pharmacist team, CPC has been an effective intervention for ID treatment in China, particularly in economically disadvantaged areas. In addition, standardized capability among clinical pharmacists with different backgrounds can be achieved in different ways. In conclusion, our study provides powerful evidence for policy/decision-makers to cognize the value of CPC and propagate this intervention mode in developing countries or regions where clinical pharmacists are insufficient in number and not actively involved in the healthcare systems. In the future, we will make effort to find a practicable way to standardize the CPC service among the different primary healthcare institutions in these areas.

## Data Availability Statement

The raw data supporting the conclusions of this article will be made available by the authors, without undue reservation.

## Ethics Statement

The studies involving human participants were reviewed and approved by the Ethics Committee of the Guizhou Provincial People’s Hospital. Written informed consent to participate in this study was provided by the participants’ legal guardian/next of kin.

## Author Contributions

JZ, LL, TL, RHu, MH, XYL, XW, QF, LW, YL, SC, and XQ collected the data. JZ and XSL involved in statistical analysis. JZ, RH, WZ, and JX drafted the manuscript. JZ, XL, RHe, and JS-WK revised the final manuscript. All authors contributed to the article and approved the submitted version.

## Funding

This work was supported by the Medical Science Research Program of Beijing Health Care Foundation (YWJKJJHKYJJ-B17444). The funder contributed to the registry database establishment. The funder had no role in the study design, data collection, data analysis, data interpretation, or writing of the report. The corresponding author had full access to all the data in the study and had final responsibility for the decision to submit for publication.

## Conflict of Interest

The authors declare that the research was conducted in the absence of any commercial or financial relationships that could be construed as a potential conflict of interest.

## References

[B1] Abubakar U. Tangiisuran B. (2020). Nationwide survey of Pharmacist’s involvement in antimicrobial stewardship program in Nigerian Tertiary Hospitals. J. Glob. Antimicrob. Resist. 21, 148–153. 10.1016/j.jgar.2019.10.007 31628999

[B2] Alsan M. Schoemaker L. Egglestn K. Kammili N. Kolli P. Bhattacharya J. (2015). Out-of-pocket health expenditures and antimicrobial resistance in low- and middle-income countries. Lancet Infect. Dis. 15, 1203–1210. 10.1016/S1473-3099(15)00149-8 26164481 PMC4609169

[B3] Bessesen M. T. Ma A. Clegg D. Fugit R. V. Pepe A. Goetz M. B. (2015). Antimicrobial Stewardship Programs: Comparison of a Program with Infectious Diseases Pharmacist Support to a Program with a Geographic Pharmacist Staffing Model. Hosp. Pharm. 50, 477–483. 10.1310/hpj5006-477 26405339 PMC4568108

[B4] Bilal A. II Tilahun Z. Gebretekle G. B. Ayalneh B. Hailemeskel B. Engidawork E. (2017). Current status, challenges and the way forward for clinical pharmacy service in Ethiopian public hospitals. BMC. Health Serv. Res. 17, 359. 10.1186/s12913-017-2305-1 28526021 PMC5437556

[B5] Bishop P. A. Isache C. McCarter Y. S. Smotherman C. Gautam S. Jankowski C. A. (2020). Clinical impact of a pharmacist-led antimicrobial stewardship initiative evaluating patients with Clostridioides difficile colitis. J. Investig. Med. 68, 888–892. 10.1136/jim-2019-001173 32066570

[B6] Brink A. J. Messina A. P. Feldman C. Richards G. A. Becker P. J. Goff D. A. (2016). Antimicrobial stewardship across 47 South African hospitals: an implementation study. Lancet Infect. Dis. 16, 1017–1025. 10.1016/S1473-3099(16)30012-3 27312577

[B7] Charani E. Smith I. Skodvin B. Perozziello A. Lucet J. C. Lescure F. X. (2019). Investigating the cultural and contextual determinants of antimicrobial stewardship programmes across low-, middle- and high-income countries-A qualitative study. PLoS One 14, e0209847. 10.1371/journal.pone.0209847 30650099 PMC6335060

[B8] EI Hajj M. S. Al-Saeed H. S. Khaja M. (2016). Qatar pharmacists’ understanding, attitudes, practice and perceived barriers related to providing pharmaceutical care. Int. J. Clin. Pharm. 38, 330–343. 10.1007/s11096-016-0246-0 26758716

[B9] Ellis K. Rubal-Peace G. Chang V. Liang E. Wong N. Campbell S. (2016). Antimicrobial stewardship for a geriatric behavioral health population. Antibiotics 5, E8. 10.3390/antibiotics5010008 27025523 PMC4810410

[B10] Fay L. N. Wolf L. M. Brandt K. L. DeYoung G. R. Anderson A. M. Egwuatu N. (2019). Pharmacist-led antimicrobial stewardship program in an urgent care setting. Am. J. Health Syst. Pharm. 76, 175–181. 10.1093/ajhp/zxy023 30689745 PMC6366123

[B11] Gebretekle G. B. Mariam D. H. Abebe W. Amogne W. Tenna A. Fenta T. G. (2018). Opportunities and barriers to implementing antibiotic stewardship in low and middle-income countries: lessons from a mixedmethods study in a tertiary care hospital in Ethiopia. PLoS One 13, e0208447. 10.1371/journal.pone.0208447 30571688 PMC6301706

[B12] Hayat K. Li P. Rosenthal M. Xu S. Chang J. Gillani A. H. (2019). Perspective of community pharmacists about community-based antimicrobial stewardship programs. A multicenter cross-sectional study from China. Expert Rev. Anti Infect. Ther. 17, 1043–1050. 10.1080/14787210.2019.1692655 31714841

[B13] Hayat K. Rosenthal M. Gillani A. H. Chang J. Ji W. Yang C. (2020). Perspective of key healthcare professionals on antimicrobial resistance and stewardship programs: a multicenter cross-sectional study from Pakistan. Front. Pharmacol. 10:1520:1520. 10.3389/fphar.2019.01520 31998124 PMC6967405

[B14] Hu F. Zhu D. Wang F. Wang M. (2018). Current status and trends of antibacterial resistance in China. Clin. Infect. Dis. 13, S128–S134. 10.1093/cid/ciy657 30423045

[B15] Katoue M. G. Awad A. II Schwinghammer T. L. Kombian S. B. (2014). Pharmaceutical care in Kuwait: hospital pharmacists’ perspectives. Int. J. Clin. Pharm. 36, 1170–1178. 10.1007/s11096-014-0013-z 25204259

[B16] Laxminarayan R. Chaudhury R. R. (2016). Antibiotic resistance in India: drivers and opportunities for action. PLoS Med. 13, e1001974. 10.1371/journal.pmed.1001974 26934098 PMC4775002

[B17] Li Z. Cheng B. Zhang K. Xie G. Wang Y. Hou J. (2017). Pharmacist-driven antimicrobial stewardship in intensive care units in East China: a multicenter prospective cohort study. Am. J. Infect. Control. 45, 983–989. 10.1016/j.ajic.2017.02.021 28596021

[B18] Li P. Hayat K. Shi L. Lambojon K. Saeed A. Aziz M. M. (2020). Knowledge, attitude, and practices of antibiotics and antibiotic resistance among Chinese pharmacy customers: a multicenter survey study. Antibiotics 9, 184. 10.3390/antibiotics9040184 PMC7235738 32316147

[B19] Marston H. D. Dixon D. M. Knisely J. M. Palmore T. N. Fauci A. S. (2016). Antimicrobial resistance. JAMA 316, 1193–1204. 10.1001/jama.2016.11764 27654605

[B20] Northey A. McGuren T. Stupans I. (2015). Patients’ antibiotic knowledge: a trial assessing the impact of verbal education. Int. J. Pharm. Pract. 23, 158–160. 10.1111/ijpp.12136 25040636

[B21] Okada N. Fushitani S. Azuma M. Nakamura S. Nakamura T. Teraoka K. (2016). Clinical evaluation of pharmacist interventions in patients treated with anti-methicillin-resistant Staphylococcus aureus agents in a hematological Ward. Bio. Pharm. Bull. 38, 295–300. 10.1248/bpb.b15-00774 26830489

[B22] O’Neill J. (2016). Tackling Drug-Resistant Infections Globally: Final Report and Recommendations. Available at: https://amr-review.org/Publications.html (Accessed May 2, 2020).

[B23] Sadyrbaeva-Dolgova S. Aznarte-Padial P. Jimenez-Morales A. Exposito-Ruiz M. Calleja-Hernandez M. A. C. Hidalgo-Tenorio C. (2020). Pharmacist recommendations for carbapenem de-escalation in urinary tract infection within an antimicrobial stewardship program. J. Infect. Public. Health 13, 558–563. 10.1016/j.jiph.2019.09.014 31685404

[B24] Salim A. M. A. Elhada A. H. A. Elgizoli B. (2016). Exploring clinical pharmacists’ perception of their impact on healthcare in Khartoum state, Sudan. J. Res. Pharm. Pract. 5, 272–278. 10.4103/2279-042X.192459 27843964 PMC5084485

[B25] Shi L. Chang J. Liu X. Zhai P. Hu S. Li P. (2020). Dispensing Antibiotics without a prescription for acute cough associated with common cold at community pharmacies in Shenyang, Northeastern China: a cross-sectional study. Antibiotics 9, 163. 10.3390/antibiotics9040163 PMC7235837 32268530

[B26] Smith J. R. Frens J. J. Snider C. B. Claeys K. C. (2018). Impact of a pharmacist-driven care package on Staphylococcus aureus bacteremia management in a large community healthcare network: A propensity score-matched, quasi-experimental study. Diagn. Microbiol. Infect. Dis. 90, 50–54. 10.1016/j.diagmicrobio.2017.10.001 29153470

[B27] Thakur S. Gray G. C. (2019). The mandate for a global one health approach to antimicrobial resistance surveillance. Am. J. Trop. Med. Hyg. 100, 227–228. 10.4269/ajtmh.18-0973 30608047 PMC6367630

[B28] Wang F. Prier B. Bauer K. A. Mellett J. (2018). Pharmacist-driven initiative for management of Staphylococcus aureus bacteremia using a clinical decision support system. Am. J. Health Syst. Pharm. 75, S35–S41. 10.2146/ajhp170087 29802177

[B29] Waters C. D. (2015). Pharmacist-driven antimicrobial stewardship program in an institution without infectious diseases physician support. Am. J. Health Syst. Pharm. 72, 466–468. 10.2146/ajhp140381 25736941

[B30] Wirtz A. L. Burns A. N. Lee B. R. Frank T. S. Fitzmaurice L. Ogden R. K. (2020). Effectiveness and safety of mandatory antimicrobial indications and durations and a pharmacist-driven 48-hour time-out in a pediatric hospital. Am. J. Health Syst. Pharm. 77, 614–621. 10.1093/ajhp/zxaa029 32236453

[B31] World Health Organization (2014). Antimicrobial resistance: global report on surveillance 2014. Available at: https://www.who.int/antimicrobial-resistance/publications/surveillancereport/en (Accessed May 2, 2020).

[B32] Zhang J. Li X. Xie J. Zheng W. (2019a). Evaluation of a clinical pharmacist consultation service for patients with infectious diseases in China: a systematic review. Eur. J. Hosp. Pharm. Sci. Pract. 0, 1–6. 10.1136/ejhpharm-2018-001815 PMC7223343 32419932

[B33] Zhang J. Qian X. Zhang L. Hu L. Fan L. Wang Q. (2019b). Evaluation of the effectiveness of clinical pharmacists’ consultation in the treatment of infectious diseases: a single-arm, prospective cohort study. Front. Pharmacol. 10:187:187. 10.3389/fphar.2019.00187 30881307 PMC6405418

[B34] Zhang J. Qian X. Xiong S. Chen Q. Bai X. Fan L. (2020a). Establishment of the templates and evaluation opinions from clinical pharmacists in the treatment of infectious diseases. Chin. J. Hosp. Pharm. 40, 708–713. 10.13286/j.1001-5213.2020.06.22

[B35] Zhang J. Xu C. Zheng W. He R. Xie J. Qian X. (2020b). The clinical pharmacist-led consultation for infectious diseases in Guizhou Province, China: a survey among hospital pharmacies. Front. Pharmacol. 11:149:149. 10.3389/fphar.2020.00149 32174837 PMC7056738

[B36] Zhou Y. Ma L. Y. Zhao X. Tian S. H. Sun L. Y. Cui Y. M. (2015). Impact of pharmacist intervention on antibiotic use and prophylactic antibiotic use in urology clean operations. J. Clin. Pharm. Ther. 40, 404–408. 10.1111/jcpt.12275 25913640

