# Supplementary material for: The Effectiveness of Clinical Pharmacist-Led Consultation in the Treatment of Infectious Diseases: A Prospective, Multicenter, Cohort Study
Source: Front Pharmacol. 2020 Sep 8;11:575022. doi: 10.3389/fphar.2020.575022 (PMC7506045; doi:10.3389/fphar.2020.575022)
Supplement: Supplementary file 1 [file DataSheet_1.doc]

Table S1 STROBE Statement—Checklist of items that should be included in reports of ***cohort studies***

|  | Item No | Recommendation | Reported on Page # |
| --- | --- | --- | --- |
| **Title and abstract** | 1 | (*a*) Indicate the study’s design with a commonly used term in the title or the abstract | Page 1 |
|  |  | (*b*) Provide in the abstract an informative and balanced summary of what was done and what was found | Page 3 |
| Introduction | | |  |
| Background/rationale | 2 | Explain the scientific background and rationale for the investigation being reported | Page 4 |
| Objectives | 3 | State specific objectives, including any prespecified hypotheses | Page 5 |
| Methods | | |  |
| Study design | 4 | Present key elements of study design early in the paper | Page 5 |
| Setting | 5 | Describe the setting, locations, and relevant dates, including periods of recruitment, exposure, follow-up, and data collection | Page 5-7 |
| Participants | 6 | (*a*) Give the eligibility criteria, and the sources and methods of selection of participants. Describe methods of follow-up | Page 6-7 |
|  |  | (*b*) For matched studies, give matching criteria and number of exposed and unexposed | Not applicable |
| Variables | 7 | Clearly define all outcomes, exposures, predictors, potential confounders, and effect modifiers. Give diagnostic criteria, if applicable | Page 6-7 |
| Data sources/ measurement | 8* | For each variable of interest, give sources of data and details of methods of assessment (measurement). Describe comparability of assessment methods if there is more than one group | Page 6-7 |
| Bias | 9 | Describe any efforts to address potential sources of bias | Page 7 |
| Study size | 10 | Explain how the study size was arrived at | Not applicable |
| Quantitative variables | 11 | Explain how quantitative variables were handled in the analyses. If applicable, describe which groupings were chosen and why | Not applicable |
| Statistical methods | 12 | (*a*) Describe all statistical methods, including those used to control for confounding | Page 8 |
|  |  | (*b*) Describe any methods used to examine subgroups and interactions | Not applicable |
|  |  | (*c*) Explain how missing data were addressed | Page 8 |
|  |  | (*d*) If applicable, explain how loss to follow-up was addressed | Not applicable |
|  |  | (*e*) Describe any sensitivity analyses | Page 8 |
| Results | | |  |
| Participants | 13* | (a) Report numbers of individuals at each stage of study—eg numbers potentially eligible, examined for eligibility, confirmed eligible, included in the study, completing follow-up, and analysed | Fig 1 and Page 8 |
|  |  | (b) Give reasons for non-participation at each stage | Fig 1 |
|  |  | (c) Consider use of a flow diagram | Fig 1 |
| Descriptive data | 14* | (a) Give characteristics of study participants (eg demographic, clinical, social) and information on exposures and potential confounders | Page 8 and Table 1 |
|  |  | (b) Indicate number of participants with missing data for each variable of interest | Not applicable |
|  |  | (c) Summarise follow-up time (eg, average and total amount) | Not applicable |
| Outcome data | 15* | Report numbers of outcome events or summary measures over time | Page 9 and Table 1 |
| Main results | 16 | (*a*) Give unadjusted estimates and, if applicable, confounder-adjusted estimates and their precision (eg, 95% confidence interval). Make clear which confounders were adjusted for and why they were included | Page 9-10  Figure 2 and Figure 3  Table S4 and Table S7 |
|  |  | (*b*) Report category boundaries when continuous variables were categorized | Not applicable |
|  |  | (*c*) If relevant, consider translating estimates of relative risk into absolute risk for a meaningful time period | Not applicable |
| Other analyses | 17 | Report other analyses done—eg analyses of subgroups and interactions, and sensitivity analyses | Page 9  Tables S5-S6  Tables S8-S9 |
| Discussion | | |  |
| Key results | 18 | Summarise key results with reference to study objectives | Page 10 |
| Limitations | 19 | Discuss limitations of the study, taking into account sources of potential bias or imprecision. Discuss both direction and magnitude of any potential bias | Page 12-13 |
| Interpretation | 20 | Give a cautious overall interpretation of results considering objectives, limitations, multiplicity of analyses, results from similar studies, and other relevant evidence | Page 11-12 |
| Generalisability | 21 | Discuss the generalisability (external validity) of the study results | Page 13 |
| Other information | | |  |
| Funding | 22 | Give the source of funding and the role of the funders for the present study and, if applicable, for the original study on which the present article is based | Page 14 |

*Give information separately for exposed and unexposed groups.

**Note:** An Explanation and Elaboration article discusses each checklist item and gives methodological background and published examples of transparent reporting. The STROBE checklist is best used in conjunction with this article (freely available on the Web sites of PLoS Medicine at http://www.plosmedicine.org/, Annals of Internal Medicine at http://www.annals.org/, and Epidemiology at http://www.epidem.com/). Information on the STROBE Initiative is available at http://www.strobe-statement.org.


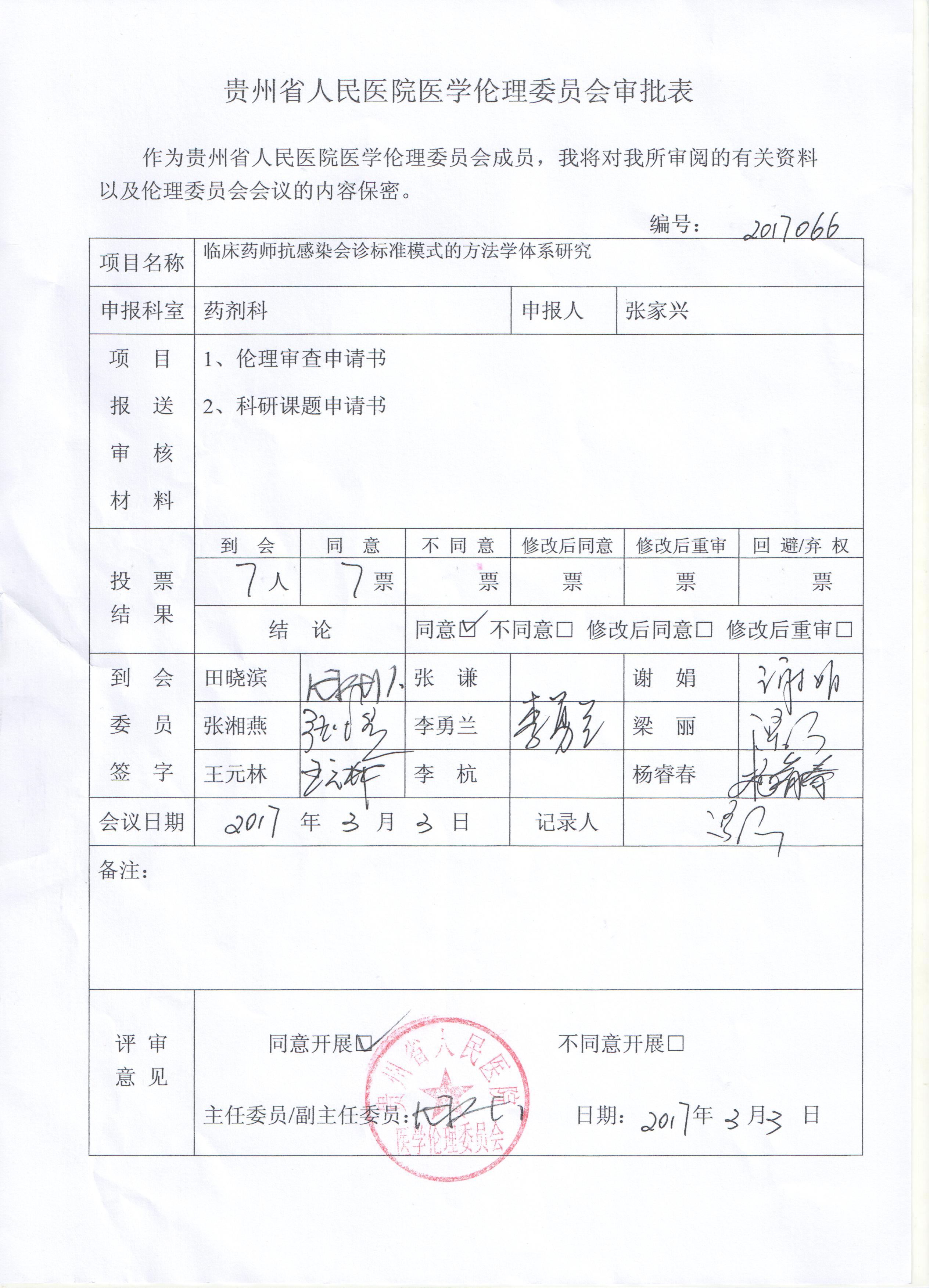
Table S2. Ethical Approval Documentation (Chinese)

Table S3. Ethical Approval Documentation (English)

**Approval form of the Medical Ethics Committee of Guizhou Provincial People’s Hospital**

As a member of the Medical Ethics Committee of Guizhou Provincial People’s Hospital, I will keep secret for the materials which I reviewed and the content of the conference.

The Number: 2017066

| Title of the project | Methodological research on the standard procedure of clinical pharmacists’ consultation for infectious diseases. | | | | | |
| --- | --- | --- | --- | --- | --- | --- |
| Department | Department of Pharmacy | | | Applicant | Zhang Jiaxing | |
| The materials for ethical review | 1. Application for ethical review  2. Application for the study projects | | | | | |
| The results of voting | Attendance  (n) | Agree  (n) | Disagree  (n) | Agree after the project was revised  (n) | Review after the project was revised  (n) | Avoidance / abstention  (n) |
|  | 7 | 7 |  |  |  |  |
|  | Conclusion | | Agree√ Disagree□  Agree after the project was revised□  Review after the project was revised□ | | | |
| Member of the ethics committee | Tian Xiaobing |  | Zhang Qian |  | Xie juan |  |
|  | Zhang Xiangyan |  | Li Yonglan |  | Liang Li |  |
|  | Wang Yuanlin |  | Li Hang |  | Yang Ruichun |  |
| Conference date | 3/3/2017 | | | Conference recorder | Wang Han | |
| Remark: None | | | | | | |
| Review comments | Agree to conduct this project√  Disagree to conduct this project□  Chairman: Date: 3/3/2017 | | | | | |

**Table S4. The results of univariate analyses for all the included patients (n=2663)**

| **Variables** | ***OR*** | ***95%CI*** | ***P value*** |
| --- | --- | --- | --- |
| **Group**  Intervention vs. Control | 2.131 | [1.466, 3.097] | < 0.001 ^*^ |
| **Sex**  Male vs. Female | 0.727 | [0.596, 0.887] | 0.002 ^*^ |
| **Age** 0-17 years vs. 18-65 years | 0.967 | [0.700, 1.336] | 0.840 |
| ≥65 years vs. 18-65 years | 0.958 | [0.775, 1.185] | 0.694 |
| **Department** Surgical Department vs. Intensive Care Unit | 5.856 | [4.324, 7.929] | < 0.001 ^*^ |
| Pediatric Department vs. Intensive Care Unit | 3.349 | [2.116, 5.301] | < 0.001 ^*^ |
| Oncology Department vs. Intensive Care Unit | 1.888 | [1.298, 2.745] | 0.001 ^*^ |
| Infection & Respiratory Department vs. Intensive Care Unit | 2.014 | [1.312, 3.094] | 0.001 ^*^ |
| Emergency Department vs. Intensive Care Unit | 3.365 | [1.875, 6.039] | < 0.001 ^*^ |
| Traditional Chinese Medicine Department vs. Intensive Care Unit | 1.074 | [0.236, 4.900] | 9.260 |
| Invasive Technology Department vs. Intensive Care Unit | 13.429 | [4.080, 44.202] | < 0.001 ^*^ |
| Internal Medicine Department vs. Intensive Care Unit | 3.757 | [2.679, 5.269] | < 0.001 ^*^ |
| **Type of consultation** Special Consultation vs. General Consultation | 0.803 | [0.525, 1.230] | 0.313 |
| **Hypoalbuminemia** Yes vs. No | 0.449 | [0.348, 0.580] | < 0.001 ^*^ |
| NA vs. No | 0.642 | [0.460, 0.898] | 0.010 ^*^ |
| **Kidney function** Abnormal vs. Normal | 0.621 | [0.504, 0.766] | < 0.001 ^*^ |
| NA vs. Normal | 1.595 | [0.918, 2.770] | 0.097 |
| **Liver function** Abnormal vs. Normal | 0.550 | [0.446, 0.678] | < 0.001 ^*^ |
| NA vs. Normal | 1.810 | [0.984, 3.329] | 0.056 |
| **With etiological evidence** Yes vs. No | 0.943 | [0.777, 1.146] | 0.556 |
| **With imaging examination** Yes vs. No | 0.476 | [0.389, 0.582] | < 0.001 ^*^ |
| **Comorbidity** Yes vs. No | 0.764 | [0.629, 0.927] | 0.006 ^*^ |
| **With high risk factors of infection** Yes vs. No | 0.555 | [0.454, 0.677] | < 0.001 ^*^ |
| **Surgical treatment of infectious sites** Yes vs. No | 1.572 | [1.223, 2.020] | < 0.001 ^*^ |
| **Severity of infection** | 0.447 | [0.382, 0.522] | < 0.001 ^*^ |
| **Time of consultation after hospitalized** ＞7 days vs. 1-7 days | 1.092 | [0.900, 1.324] | 0.373 |
| **Purpose of consultation** Initial Therapeutic Regimen vs. Therapeutic Regimen Adjustment | 1.629 | [1.164, 2.282] | 0.004 ^*^ |
| **Number of infectious sites** ＞1 vs. 1 | 0.727 | [0.566, 0.934] | 0.013 ^*^ |
| NA vs. 1 | 0.531 | [0.358, 0.788] | 0.002 ^*^ |
| **Febrile symptom**  Yes vs. No | 0.564 | [0.463, 0.688] | < 0.001 ^*^ |
| **Hemogram** Abnormal vs. Normal | 0.657 | [0.503, 0.859] | 0.002 ^*^ |
| NA vs. Normal | 0.731 | [0.352, 1.519] | 0.402 |
| **Inflammatory indicator**  Increased vs. Normal | 0.459 | [0.340, 0.621] | < 0.001 ^*^ |
| NA vs. Normal | 0.708 | [0.492, 1.017] | 0.062 |
| **Type of infection**  Hospital-acquired infection vs. Community-acquired infection | 1.036 | [0.832, 1.291] | 0.752 |
| NA vs. Community-acquired infection | 1.974 | [1.354, 2.877] | < 0.001 ^*^ |

**OR=Odds Ratio; CI=Confidence Interval; ^*^=indicated P ≤0.05; NA= Not Applicable; Control group: patients in whom treatment regimens did not follow clinical pharmacist’s recommendations; Intervention group: patients in whom treatments adhere to clinical pharmacist’s recommendations.**

**Table S5. The results of sensitivity analyses using a random intercept model for all the included patients (n=2663)**

| **Variables** | ***Adjusted OR*** | ***95%CI*** | ***P value*** |
| --- | --- | --- | --- |
| **Group**  Intervention vs. Control | 1.838 | [1.212, 2.786] | 0.004 ^*^ |
| **Sex**  Male vs. Female | 0.801 | [0.646, 0.994] | 0.043 ^*^ |
| **Age** 0-17 years vs. 18-65 years | 1.660 | [0.984, 2.800] | 0.058 |
| ≥66 years vs. 18-65 years | 1.090 | [0.855, 1.390] | 0.486 |
| **Department** Surgical Department vs. Intensive Care Unit | 3.441 | [2.409, 4.916] | < 0.001 ^*^ |
| Pediatric Department vs. Intensive Care Unit | 1.850 | [0.977, 3.503] | 0.059 |
| Oncology Department vs. Intensive Care Unit | 1.753 | [1.154, 2.663] | 0.009 ^*^ |
| Infection & Respiratory Department vs. Intensive Care Unit | 2.155 | [1.355, 3.427] | 0.001 ^*^ |
| Emergency Department vs. Intensive Care Unit | 3.034 | [1.623, 5.670] | 0.001 ^*^ |
| Traditional Chinese Medicine Department vs. Intensive Care Unit | 0.856 | [0.169, 4.331] | 0.851 |
| Invasive Technology Department vs. Intensive Care Unit | 5.540 | [1.549, 19.822] | 0.008 ^*^ |
| Internal Medicine Department vs. Intensive Care Unit | 2.557 | [1.725, 3.792] | < 0.001 ^*^ |
| **Type of consultation** Special Consultation vs. General Consultation | 1.832 | [1.106, 3.035] | 0.019 ^*^ |
| **Hypoalbuminemia** Yes vs. No | 0.694 | [0.523, 0.921] | 0.012 ^*^ |
| NA vs. No | 0.768 | [0.527, 1.120] | 0.171 |
| **Kidney function** Abnormal vs. Normal | 0.901 | [0.708, 1.147] | 0.398 |
| NA vs. Normal | 0.848 | [0.298, 2.415] | 0.758 |
| **Liver function** Abnormal vs. Normal | 0.705 | [0.559, 0.889] | 0.003 ^*^ |
| NA vs. Normal | 1.899 | [0.603, 5.979] | 0.273 |
| **With etiological evidence** Yes vs. No | 0.956 | [0.770, 1.187] | 0.686 |
| **With imaging examination** Yes vs. No | 0.775 | [0.608, 0.987] | 0.039 ^*^ |
| **Coexisting disorder** Yes vs. No | 0.920 | [0.731, 1.157] | 0.475 |
| **With high risk factors of infection** Yes vs. No | 0.775 | [0.613, 0.980] | 0.033 ^*^ |
| **Surgical treatment of infectious sites** Yes vs. No | 1.380 | [1.039, 1.834] | 0.026 ^*^ |
| **Severity of infection** | 0.631 | [0.529, 0.753] | < 0.001 ^*^ |
| **Constant** | 6.518 | [3.049, 13.930] | < 0.001 ^*^ |
| **Random-effects Parameters** Variance (constant) | 0.178 | [0.060, 0.529] | - |

**OR=Odds Ratio; CI=Confidence Interval; ^*^=indicated P ≤0.05; NA= Not Applicable; Control group: patients in whom treatment regimens did not follow clinical pharmacist’s recommendations; Intervention group: patients in whom treatments adhere to clinical pharmacist’s recommendations.**

**Table S6. The results of sensitivity analyses using a random coefficient model involving all the covariates for all the included patients (n=2663)**

| **Variables** | ***OR*** | ***95%CI*** | ***P value*** |
| --- | --- | --- | --- |
| **Group**  Intervention vs. Control | 2.351 | [1.457, 3.792] | < 0.001 ^*^ |
| **Sex**  Male vs. Female | 0.791 | [0.636, 0.982] | 0.034 ^*^ |
| **Age** 0-17 years vs. 18-65 years | 1.581 | [0.935, 2.672] | 0.088 |
| ≥65 years vs. 18-65 years | 1.065 | [0.833, 1.362] | 0.613 |
| **Department** Surgical Department vs. Intensive Care Unit | 3.416 | [2.382, 4.899] | < 0.001 ^*^ |
| Pediatric Department vs. Intensive Care Unit | 1.735 | [0.908, 3.313] | 0.095 |
| Oncology Department vs. Intensive Care Unit | 1.859 | [1.195, 2.894] | 0.006 ^*^ |
| Infection & Respiratory Department vs. Intensive Care Unit | 2.180 | [1.363, 3.486] | 0.001 ^*^ |
| Emergency Department vs. Intensive Care Unit | 2.950 | [1.572, 5.534] | 0.001 ^*^ |
| Traditional Chinese Medicine Department vs. Intensive Care Unit | 0.836 | [0.165, 4.227] | 0.828 |
| Invasive Technology Department vs. Intensive Care Unit | 5.636 | [1.568, 20.260] | 0.008 ^*^ |
| Internal Medicine Department vs. Intensive Care Unit | 2.569 | [1.727, 3.821] | < 0.001 ^*^ |
| **Type of consultation** Special Consultation vs. General Consultation | 1.710 | [1.024, 2.856] | 0.040 ^*^ |
| **Hypoalbuminemia** Yes vs. No | 0.725 | [0.543, 0.968] | 0.029 ^*^ |
| NA vs. No | 0.789 | [0.536, 1.159] | 0.227 |
| **Kidney function** Abnormal vs. Normal | 0.920 | [0.721, 1.174] | 0.505 |
| NA vs. Normal | 0.812 | [[0.286, 2.307] | 0.696 |
| **Liver function** Abnormal vs. Normal | 0.718 | [0.568, 0.907] | 0.005 ^*^ |
| NA vs. Normal | 2.082 | [0.661, 6.561] | 0.210 |
| **With etiological evidence** Yes vs. No | 0.924 | [0.740, 1.152] | 0.482 |
| **With imaging examination** Yes vs. No | 0.760 | [0.594, 0.973] | 0.029 ^*^ |
| **Coexisting disorder** Yes vs. No | 0.922 | [0.732, 1.163] | 0.495 |
| **With high risk factors of infection** Yes vs. No | 0.780 | [0.616, 0.987] | 0.039 ^*^ |
| **Surgical treatment of infectious sites** Yes vs. No | 1.362 | [1.019, 1.819] | 0.037 ^*^ |
| **Severity of infection** | 0.644 | [0.533, 0.778] | < 0.001 ^*^ |
| **Time of consultation after hospitalized** ＞7 days vs. 1-7 days | 1.056 | [0.842, 1.324] | 0.637 |
| **Purpose of consultation** Initial Therapeutic Regimen vs. Therapeutic Regimen Adjustment | 1.339 | [0.920, 1.948] | 0.127 |
| **Number of infectious sites** ＞1 vs. 1 | 1.058 | [0.792, 1.412] | 0.703 |
| NA vs. 1 | 0.636 | [0.406, 0.999] | 0.049 ^*^ |
| **Febrile symptom**  Yes vs. No | 0.924 | [0.731, 1.168] | 0.507 |
| **Hemogram** Abnormal vs. Normal | 1.031 | [0.761, 1.397] | 0.845 |
| NA vs. Normal | 0.696 | [0.308, 1.574] | 0.384 |
| **Inflammatory indicator**  Increased vs. Normal | 0.631 | [0.451, 0.882] | 0.007 ^*^ |
| NA vs. Normal | 0.756 | [0.501, 1.142] | 0.184 |
| **Type of infection**  Hospital-acquired infection vs. Community-acquired infection | 0.958 | [0.733, 1.251] | 0.752 |
| NA vs. Community-acquired infection | 0.922 | [0.596, 1.428] | 0.717 |
| **Constant** | 6.796 | [2.682, 17.507] | < 0.001 ^*^ |
| **Random-effects Parameters**  Variance (group) | 0.170 | [0.056, 0.514] | - |
| Variance (constant) | 1.76×10^-11^ | 0 | - |

**OR=Odds Ratio; CI=Confidence Interval; ^*^=indicated P ≤0.05; NA= Not Applicable; Control group: patients in whom treatment regimens did not follow clinical pharmacist’s recommendations; Intervention group: patients in whom treatments adhere to clinical pharmacist’s recommendations.**

**Table S7. The results of univariate analyses for the patients in the intervention group (n=2529)**

| **Characteristic** | ***OR*** | ***95%CI*** | ***P*** |
| --- | --- | --- | --- |
| **Background of clinical pharmacist** ID vs. non-ID clinical pharmacist | 0.987 | [0.807, 1.206] | 0.896 |
| **Sex**  Male vs. Female | 0.757 | [0.616, 0.931] | 0.008 ^*^ |
| **Age** 0-17 years vs. 18-65 years | 0.916 | [0.656, 1.278] | 0.605 |
| ≥65 years vs. 18-65 years | 0.933 | [0.748, 1.163] | 0.539 |
| **Department** Surgical Department vs. Intensive Care Unit | 5.725 | [4.186, 7.830] | < 0.001 ^*^ |
| Pediatric Department vs. Intensive Care Unit | 3.264 | [2.023, 5.266] | < 0.001 ^*^ |
| Oncology Department vs. Intensive Care Unit | 1.824 | [1.242, 2.680] | 0.002 ^*^ |
| Infection & Respiratory Department vs. Intensive Care Unit | 2.078 | [1.329, 3.249] | 0.001 ^*^ |
| Emergency Department vs. Intensive Care Unit | 2.851 | [1.576, 5.158] | 0.001 ^*^ |
| Traditional Chinese Medicine Department vs. Intensive Care Unit | 1.010 | [0.221, 4.611] | 0.990 |
| Invasive Technology Department vs. Intensive Care Unit | 12.622 | [3.829, 41.609] | < 0.001 ^*^ |
| Internal Medicine Department vs. Intensive Care Unit | 3.874 | [2.723, 5.513] | < 0.001 ^*^ |
| **Type of consultation** Special Consultation vs. General Consultation | 0.756 | [0.493, 1.159] | 0.200 |
| **Hypoalbuminemia** Yes vs. No | 0.465 | [0.357, 0.606] | < 0.001 ^*^ |
| NA vs. No | 0.614 | [0.436, 0.865] | 0.005 ^*^ |
| **Kidney function** Abnormal vs. Normal | 0.662 | [0.532, 0.825] | < 0.001 ^*^ |
| NA vs. Normal | 1.512 | [0.869, 2.630] | 0.143 |
| **Liver function** Abnormal vs. Normal | 0.565 | [0.454, 0.702] | < 0.001 ^*^ |
| NA vs. Normal | 1.690 | [0.917, 3.113] | 0.092 ^*^ |
| **With etiological evidence** Yes vs. No | 0.986 | [0.804, 1.208] | 0.890 |
| **With imaging examination** Yes vs. No | 0.462 | [0.375, 0.570] | < 0.001 ^*^ |
| **Comorbidity**  Yes vs. No | 0.768 | [0.628, 0.940] | 0.010 ^*^ |
| **With high risk factors of infection** Yes vs. No | 0.521 | [0.423, 0.642] | < 0.001 ^*^ |
| **Surgical treatment of infectious sites** Yes vs. No | 1.569 | [1.209, 2.037] | 0.001 ^*^ |
| **Severity of infection** | 0.445 | [0.378, 0.524] | < 0.001 ^*^ |
| **Time of consultation after hospitalized** ＞7 days vs. 1-7 days | 1.087 | [0.9889, 1.328] | 0.417 |
| **Purpose of consultation** Initial Therapeutic Regimen vs. Therapeutic Regimen Adjustment | 1.514 | [1.075, 2.132] | 0.017 ^*^ |
| **Number of infectious sites** ＞1 vs. 1 | 0.666 | [0.514, 0.862] | 0.002 ^*^ |
| NA vs. 1 | 0.481 | [0.322, 0.719] | < 0.001 ^*^ |
| **Febrile symptom**  Yes vs. No | 0.545 | [0.443, 0.670] | < 0.001 ^*^ |
| **Hemogram** Abnormal vs. Normal | 0.634 | [0.479, 0.841] | 0.002 ^*^ |
| NA vs. Normal | 0.597 | [0.284, 1.256] | 0.174 |
| **Inflammatory indicator**  Increased vs. Normal | 0.493 | [0.362, 0.672] | < 0.001 ^*^ |
| NA vs. Normal | 0.718 | [0.496, 1.040] | 0.080 |
| **Type of infection**  Hospital-acquired infection vs. Community-acquired infection | 1.010 | [0.804, 1.268] | 0.934 |
| NA vs. Community-acquired infection | 2.120 | [1.425, 3.174] | < 0.001 ^*^ |

**ID=Infectious Diseases; OR=Odds Ratio; CI=Confidence Interval; ^*^=indicated P ≤0.05; NA= Not Applicable.**

**Table S8. The results of sensitivity analyses using a random intercept model for the patients in the intervention group (n=2529)**

| **Variables** | ***OR*** | ***95%CI*** | ***P*** |
| --- | --- | --- | --- |
| **Background of clinical pharmacist**  ID vs. non-ID clinical pharmacist | 0.958 | [0.740, 1.240] | 0.742 |
| **Sex**  Male vs. Female | 0.822 | [0.658, 1.027] | 0.084 |
| **Age** 0-17 years vs. 18-65 years | 1.550 | [0.909, 2.640] | 0.107 |
| ≥65 years vs. 18-65 years | 1.098 | [0.854, 1.412] | 0.465 |
| **Department** Surgical Department vs. Intensive Care Unit | 3.255 | [2.252, 4.704] | < 0.001 ^*^ |
| Pediatric Department vs. Intensive Care Unit | 1.948 | [1.008, 3.767] | 0.047 ^*^ |
| Oncology Department vs. Intensive Care Unit | 1.690 | [1.098, 2.600] | 0.017 ^*^ |
| Infection & Respiratory Department vs. Intensive Care Unit | 2.179 | [1.347, 3.524] | 0.001 ^*^ |
| Emergency Department vs. Intensive Care Unit | 2.286 | [1.212, 4.311] | 0.011 ^*^ |
| Traditional Chinese Medicine Department vs. Intensive Care Unit | 0.780 | [0.154, 3.954] | 0.764 |
| Invasive Technology Department vs. Intensive Care Unit | 5.698 | [1.586, 20.463] | 0.008 ^*^ |
| Internal Medicine Department vs. Intensive Care Unit | 2.607 | [1.726, 3.939] | < 0.001 ^*^ |
| **Type of consultation** Special Consultation vs. General Consultation | 1.768 | [1.064, 2.936] | 0.028 ^*^ |
| **Hypoalbuminemia** Yes vs. No | 0.719 | [0.537, 0.962] | 0.026 ^*^ |
| NA vs. No | 0.726 | [0.493, 1.068] | 0.104 |
| **Kidney function** Abnormal vs. Normal | 0.919 | [0.715, 1.181] | 0.509 |
| NA vs. Normal | 0.830 | [0.290, 2.370] | 0.727 |
| **Liver function** Abnormal vs. Normal | 0.730 | [0.574, 0.929] | 0.011 ^*^ |
| NA vs. Normal | 1.875 | [0.592, 5.943] | 0.285 |
| **With etiological evidence** Yes vs. No | 0.980 | [0.782, 1.227] | 0.860 |
| **With imaging examination** Yes vs. No | 0.741 | [0.576, 0.954] | 0.020 ^*^ |
| **Coexisting disorder** Yes vs. No | 0.956 | [0.753, 1.213] | 0.710 |
| **With high risk factors of infection** Yes vs. No | 0.710 | [0.556, 0.907] | 0.006 ^*^ |
| **Surgical treatment of infectious sites** Yes vs. No | 1.414 | [1.054, 1.897] | 0.021 ^*^ |
| **Severity of infection** | 0.643 | [0.534, 0.774] | < 0.001 ^*^ |
| **Constant** | 12.131 | [6.057, 24.295] | < 0.001 ^*^ |
| **Random-effects Parameters** Variance (constant) | 0.170 | [0.056, 0.516] | - |

**ID=Infectious Diseases; OR=Odds Ratio; CI=Confidence Interval; ^*^=indicated P ≤0.05; NA= Not Applicable.**

**Table S9. The results of sensitivity analyses using a random coefficient model involving all the covariates for the patients in the intervention group (n=2529)**

| **Variables** | ***OR*** | ***95%CI*** | ***P*** |
| --- | --- | --- | --- |
| **Background of clinical pharmacist** ID vs. non-ID clinical pharmacist | 0.971 | [0.673, 1.403] | 0.877 |
| **Sex**  Male vs. Female | 0.808 | [0.645, 1.011] | 0.062 |
| **Age** 0-17 years vs. 18-65 years | 1.435 | [0.840, 2.451] | 0.186 |
| ≥65 years vs. 18-65 years | 1.070 | [0.830, 1.379] | 0.603 |
| **Department** Surgical Department vs. Intensive Care Unit | 3.264 | [2.247, 4.739] | < 0.001 ^*^ |
| Pediatric Department vs. Intensive Care Unit | 1.770 | [0.905, 3.461] | 0.095 |
| Oncology Department vs. Intensive Care Unit | 1.854 | [1.172, 2.931] | 0.008 ^*^ |
| Infection & Respiratory Department vs. Intensive Care Unit | 2.180 | [1.338, 3.550] | 0.002 ^*^ |
| Emergency Department vs. Intensive Care Unit | 2.280 | [1.204, 4.317] | 0.011 ^*^ |
| Traditional Chinese Medicine Department vs. Intensive Care Unit | 0.767 | [0.151, 3.889] | 0.749 |
| Invasive Technology Department vs. Intensive Care Unit | 5.779 | [1.590, 21.012] | 0.008 ^*^ |
| Internal Medicine Department vs. Intensive Care Unit | 2.633 | [1.738, 3.987] | < 0.001 ^*^ |
| **Type of consultation** Special Consultation vs. General Consultation | 1.696 | [1.014, 2.838] | 0.044 ^*^ |
| **Hypoalbuminemia** Yes vs. No | 0.746 | [0.553, 1.005] | 0.054 |
| NA vs. No | 0.762 | [0.513, 1.133] | 0.180 |
| **Kidney function** Abnormal vs. Normal | 0.937 | [0.727, 1.208] | 0.615 |
| NA vs. Normal | 0.740 | [0.255, 2.148] | 0.580 |
| **Liver function** Abnormal vs. Normal | 0.743 | [0.583, 0.948] | 0.017 ^*^ |
| NA vs. Normal | 2.282 | [0.702, 7.416] | 0.170 |
| **With etiological evidence** Yes vs. No | 0.941 | [0.748, 1.185] | 0.605 |
| **With imaging examination** Yes vs. No | 0.705 | [0.543, 0.915] | 0.009 ^*^ |
| **Coexisting disorder** Yes vs. No | 0.954 | [0.750, 1.215] | 0.705 |
| **With high risk factors of infection** Yes vs. No | 0.716 | [0.559, 0.917] | 0.008 ^*^ |
| **Surgical treatment of infectious sites** Yes vs. No | 1.415 | [1.048, 1.909] | 0.023 ^*^ |
| **Severity of infection** | 0.664 | [0.544, 0.810] | < 0.001 ^*^ |
| **Time of consultation after hospitalized** ＞7 days vs. 1-7 days | 1.036 | [0.819, 1.311] | 0.769 |
| **Purpose of consultation** Initial Therapeutic Regimen vs. Therapeutic Regimen Adjustment | 1.258 | [0.858, 1.842] | 0.239 |
| **Number of infectious sites** ＞1 vs. 1 | 0.981 | [0.728, 1.323] | 0.902 |
| NA vs. 1 | 0.586 | [0.367, 0.935] | 0.025 ^*^ |
| **Febrile symptom**  Yes vs. No | 0.872 | [0.683, 1.112] | 0.269 |
| **Hemogram** Abnormal vs. Normal | 0.986 | [0.718, 1.354] | 0.932 |
| NA vs. Normal | 0.552 | [0.240, 1.273] | 0.163 |
| **Inflammatory indicator**  Increased vs. Normal | 0.657 | [0.466, 0.926] | 0.016 ^*^ |
| NA vs. Normal | 0.767 | [0.503, 1.170] | 0.218 |
| **Type of infection**  Hospital-acquired infection vs. Community-acquired infection | 0.972 | [0.738, 1.280] | 0.839 |
| NA vs. Community-acquired infection | 0.968 | [0.606, 1.545] | 0.891 |
| **Constant** | 17.058 | [7.773, 37.432] | < 0.001 ^*^ |
| **Random-effects Parameters**  Variance (major of clinical pharmacist) | 0.121 | [0.010, 1.445] | - |
| Variance (constant) | 0.078 | [0.008, 0.776] | - |

**ID=Infectious Diseases; OR=Odds Ratio; CI=Confidence Interval; ^*^=indicated P ≤0.05; NA= Not Applicable.**
